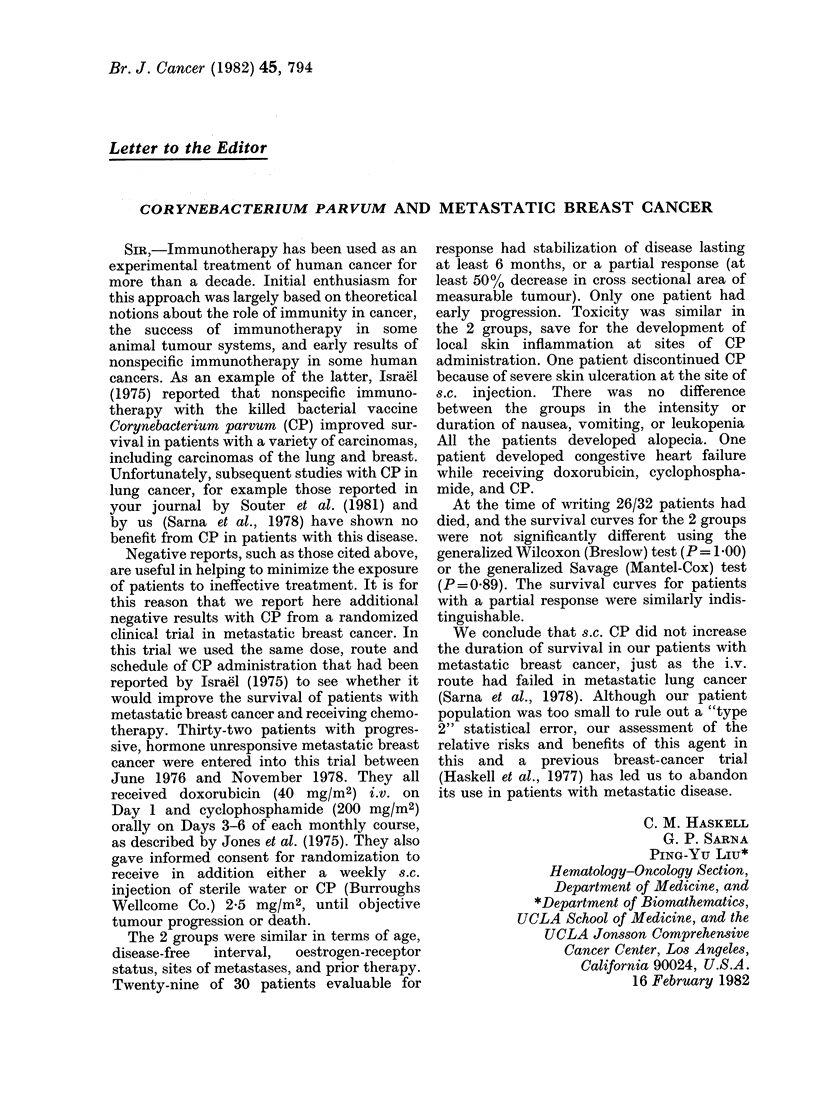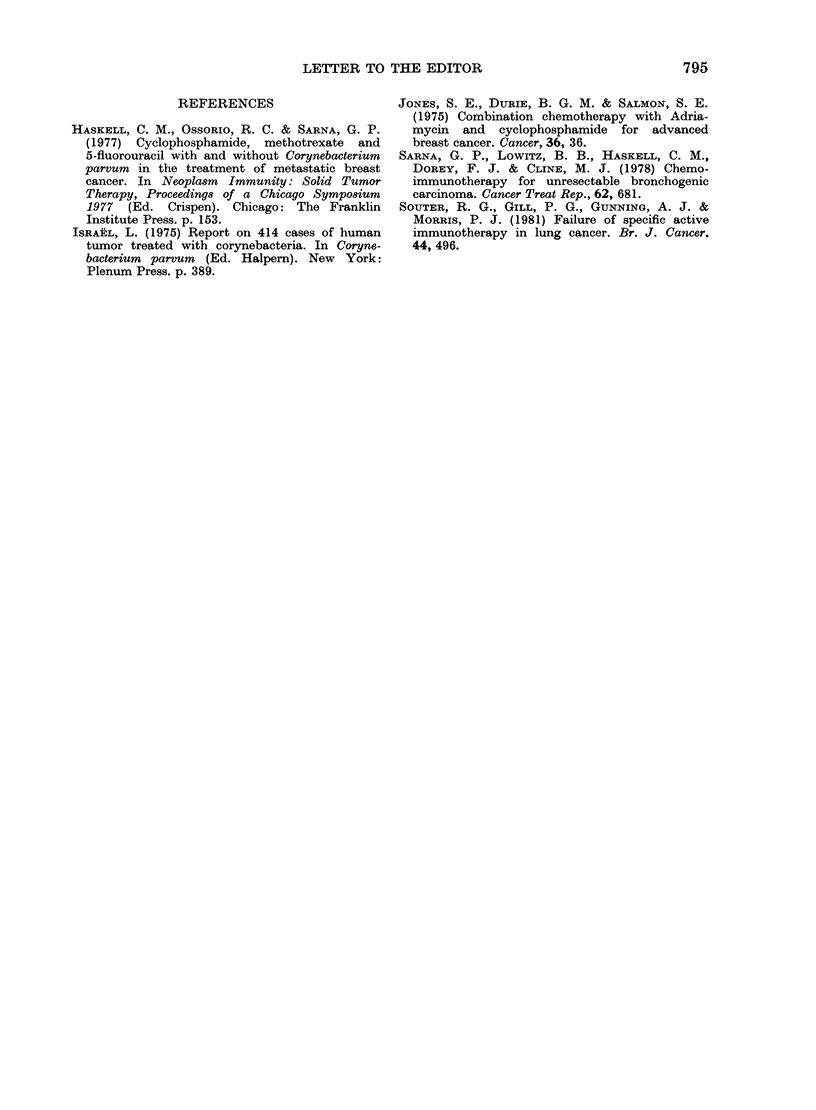# Corynebacterium parvum and metastatic breast cancer.

**DOI:** 10.1038/bjc.1982.124

**Published:** 1982-05

**Authors:** C. M. Haskell, G. P. Sarna, P. Y. Liu


*Br. J. Cancer* (1982) **45**, 794

### Letter to the Editor

### *CORYNEBACTERIUM PARVUM* AND METASTATIC BREAST CANCER

SIR,—Immunotherapy has been used as an experimental treatment of human cancer for more than a decade. Initial enthusiasm for this approach was largely based on theoretical notions about the role of immunity in cancer, the success of immunotherapy in some animal tumour systems, and early results of nonspecific immunotherapy in some human cancers. As an example of the latter, Israël (1975) reported that nonspecific immunotherapy with the killed bacterial vaccine *Corynebacterium parvum* (CP) improved survival in patients with a variety of carcinomas, including carcinomas of the lung and breast. Unfortunately, subsequent studies with CP in lung cancer, for example those reported in your journal by Souter *et al.* (1981) and by us (Sarna *et al.*, 1978) have shown no benefit from CP in patients with this disease.

Negative reports, such as those cited above, are useful in helping to minimize the exposure of patients to ineffective treatment. It is for this reason that we report here additional negative results with CP from a randomized clinical trial in metastatic breast cancer. In this trial we used the same dose, route and schedule of CP administration that had been reported by Israël (1975) to see whether it would improve the survival of patients with metastatic breast cancer and receiving chemotherapy. Thirty-two patients with progressive, hormone unresponsive metastatic breast cancer were entered into this trial between June 1976 and November 1978. They all received doxorubicin (40 mg/m$^2$) *i.v.* on Day 1 and cyclophosphamide (200 mg/m$^2$) orally on Days 3–6 of each monthly course, as described by Jones *et al.* (1975). They also gave informed consent for randomization to receive in addition either a weekly *s.c.* injection of sterile water or CP (Burroughs Wellcome Co.) 2·5 mg/m$^2$, until objective tumour progression or death.

The 2 groups were similar in terms of age, disease-free interval, oestrogen-receptor status, sites of metastases, and prior therapy. Twenty-nine of 30 patients evaluable for response had stabilization of disease lasting at least 6 months, or a partial response (at least 50% decrease in cross sectional area of measurable tumour). Only one patient had early progression. Toxicity was similar in the 2 groups, save for the development of local skin inflammation at sites of CP administration. One patient discontinued CP because of severe skin ulceration at the site of *s.c.* injection. There was no difference between the groups in the intensity or duration of nausea, vomiting, or leukopenia All the patients developed alopecia. One patient developed congestive heart failure while receiving doxorubicin, cyclophosphamide, and CP.

At the time of writing 26/32 patients had died, and the survival curves for the 2 groups were not significantly different using the generalized Wilcoxon (Breslow) test ($P=1{\cdot}00$) or the generalized Savage (Mantel-Cox) test ($P=0{\cdot}89$). The survival curves for patients with a partial response were similarly indistinguishable.

We conclude that *s.c.* CP did not increase the duration of survival in our patients with metastatic breast cancer, just as the i.v. route had failed in metastatic lung cancer (Sarna *et al.*, 1978). Although our patient population was too small to rule out a "type 2" statistical error, our assessment of the relative risks and benefits of this agent in this and a previous breast-cancer trial (Haskell *et al.*, 1977) has led us to abandon its use in patients with metastatic disease.

C. M. HASKELL
G. P. SARNA
PING-YU LIU*
*Hematology-Oncology Section,*
*Department of Medicine, and*
**Department of Biomathematics,*
*UCLA School of Medicine, and the*
*UCLA Jonsson Comprehensive*
*Cancer Center, Los Angeles,*
*California* 90024, *U.S.A.*
16 *February* 1982

### REFERENCES

HASKELL, C. M., OSSORIO, R. C. & SARNA, G. P. (1977) Cyclophosphamide, methotrexate and 5-fluorouracil with and without *Corynebacterium parvum* in the treatment of metastatic breast cancer. In *Neoplasm Immunity: Solid Tumor Therapy, Proceedings of a Chicago Symposium 1977* (Ed. Crispen). Chicago: The Franklin Institute Press. p. 153.

ISRAËL, L. (1975) Report on 414 cases of human tumor treated with corynebacteria. In *Corynebacterium parvum* (Ed. Halpern). New York: Plenum Press. p. 389.

JONES, S. E., DURIE, B. G. M. & SALMON, S. E. (1975) Combination chemotherapy with Adriamycin and cyclophosphamide for advanced breast cancer. *Cancer*, **36**, 36.

SARNA, G. P., LOWITZ, B. B., HASKELL, C. M., DOREY, F. J. & CLINE, M. J. (1978) Chemoimmunotherapy for unresectable bronchogenic carcinoma. *Cancer Treat Rep.*, **62**, 681.

SOUTER, R. G., GILL, P. G., GUNNING, A. J. & MORRIS, P. J. (1981) Failure of specific active immunotherapy in lung cancer. *Br. J. Cancer*, **44**, 496.